# Supplementary material for: Cryptic pocket formation underlies allosteric modulator selectivity at muscarinic GPCRs
Source: Nat Commun. 2019 Jul 23;10:3289. doi: 10.1038/s41467-019-11062-7 (PMC6650467; doi:10.1038/s41467-019-11062-7)
Supplement: Supplementary file 1 — Supplementary Information [file 41467_2019_11062_MOESM1_ESM.pdf]

Supplementary Information

Cryptic pocket formation underlies allosteric modulator selectivity at muscarinic GPCRs

S.A. Hollingsworth et al.

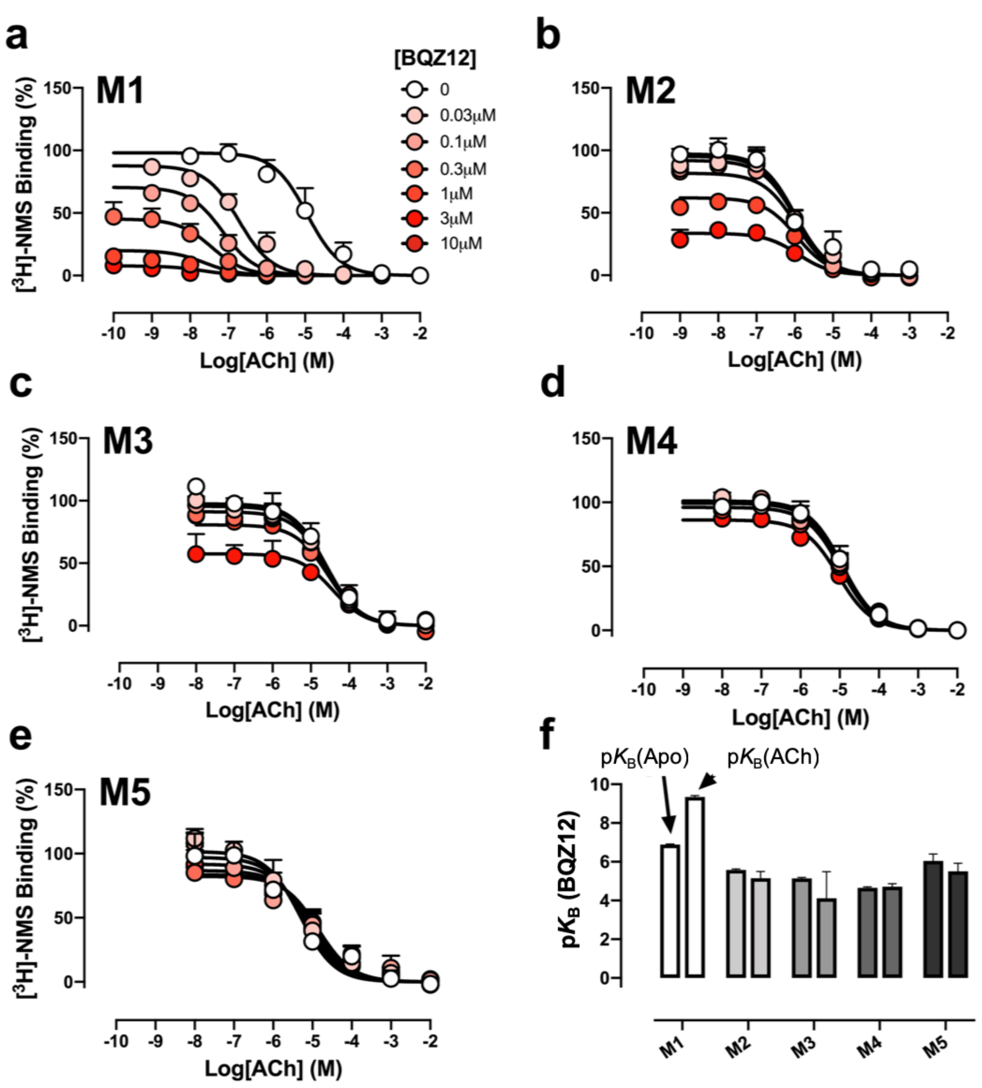

Supplementary Figure 1. Allosteric effect of BQZ12 at the M1–M5 mAChRs. Interaction radioligand binding experiments between [^{3}H]NMS and ACh in the absence or presence of increasing concentrations of BQZ12 at the M1 (**a**), M2 (**b**), M3 (**c**), M4 (**d**) and M5 (**e**) mAChRs. Data points represent the mean, and error bars the standard error of the mean, across 3 independent experiments (except M1 and M4, which represent 4 independent experiments) with each point determined in duplicate per experiment. (**f**) Quantification of BQZ12 affinity estimates for each mAChR subtype at the unoccupied receptor (pK_B(Apo), left-hand bar in each pair) and ACh-occupied receptor (pK_B(ACh), right-hand bar in each pair). Affinity estimates and their standard errors (shown by error bars) were derived by a global least-squares fit of an allosteric ternary

complex model to 3 (M2, M3, and M5) or 4 (M1 and M4) independent experiments, with the constraint that each parameter be shared between the experiments to yield a single best-estimate of the parameter and its associated standard error, as derived from the nonlinear regression algorithm. This global pooled analysis approach ensured model convergence in all instances. Source data are provided in a Source Data file.

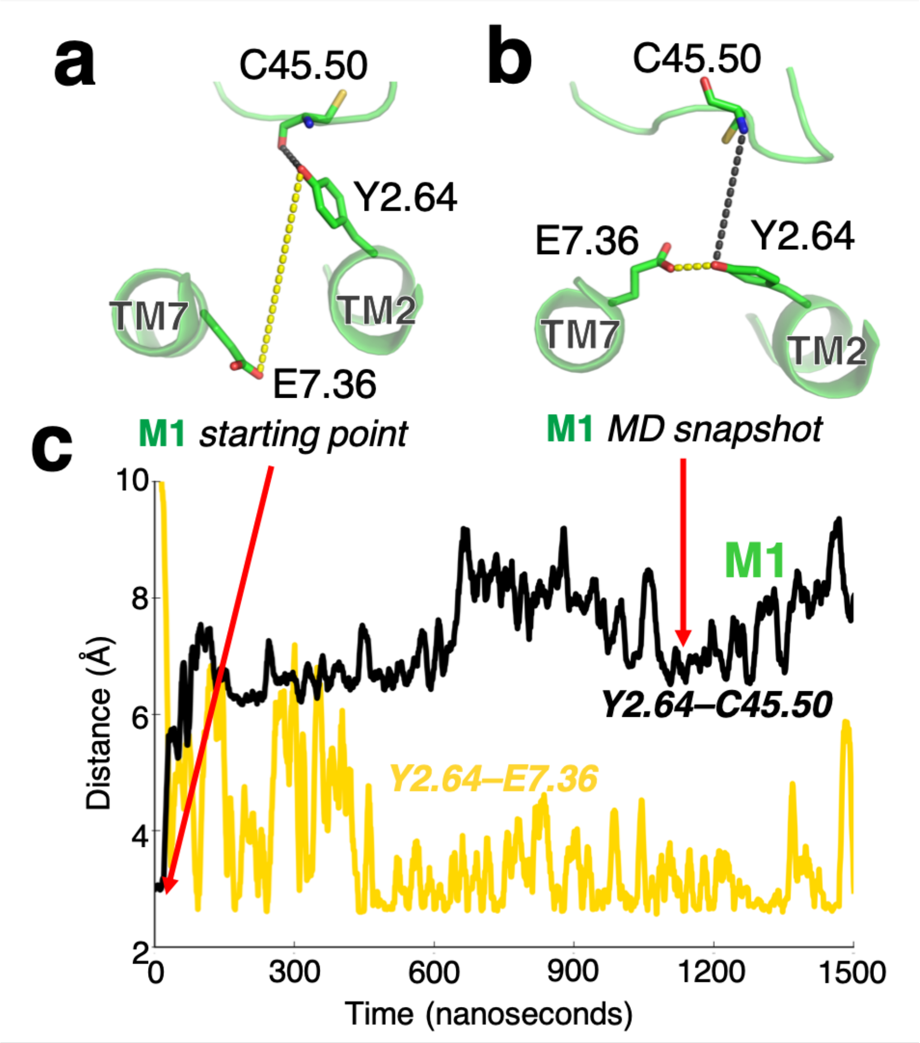

Supplementary Figure 2. Breakage of Y2.64–C45.50 hydrogen bond is followed by formation of Y2.64–E7.36 hydrogen bond. In simulations of the M1 mAChR, Y2.64 was observed to dynamically rotate away from the backbone of C45.50 (**a**) to interact with E7.36 (**b**). This rotation opens a cryptic pocket in the M1 mAChR allosteric binding site. The distance between Y2.64 and C45.50 from a representative simulation of M1 mAChR tracks the dynamic opening and closing of the cryptic pocket (**c**). This simulation was of an active-state receptor with neither orthosteric nor allosteric ligand bound, but inactive-state simulations and simulations with orthosteric ligands bound yielded similar results (Fig. 3).

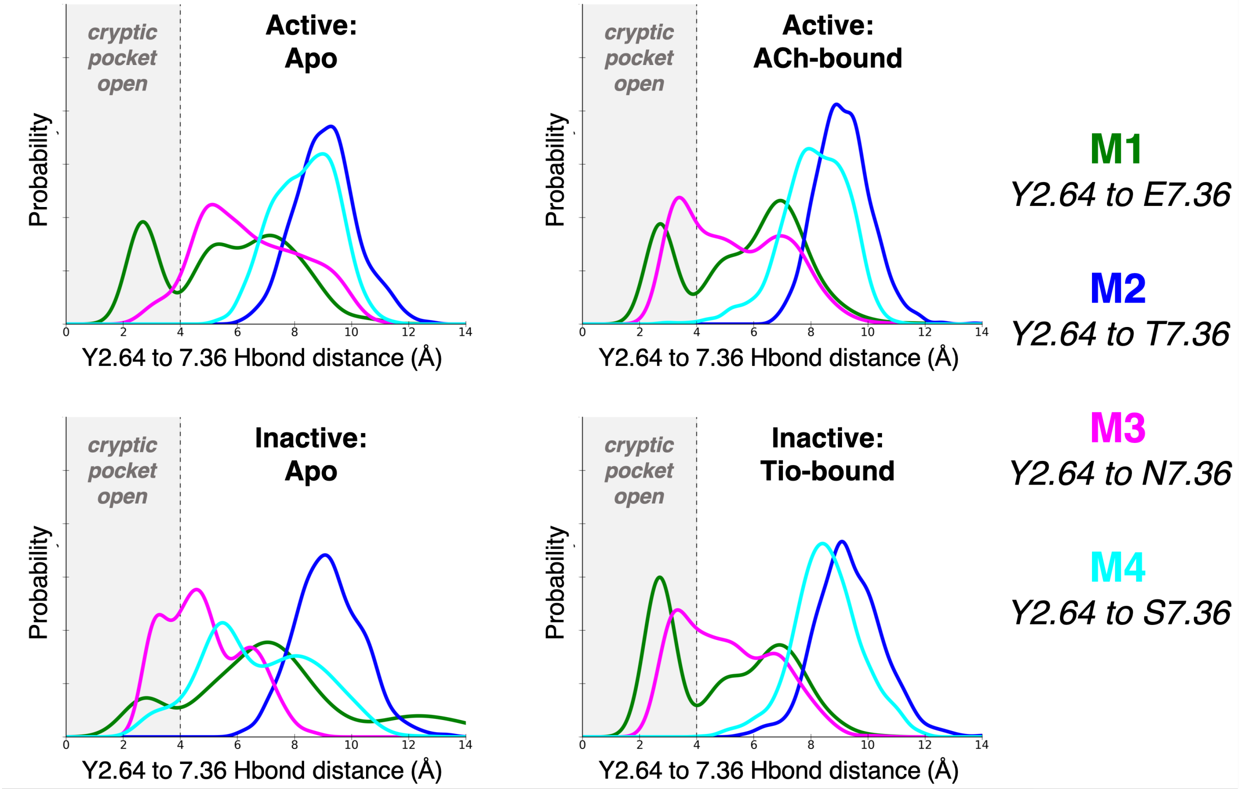

Supplementary Figure 3. Formation of the 2.64–7.36 hydrogen bond correlates with opening of the cryptic pocket. Distance distributions for the shortest potential hydrogen bond (Hbond) between the side chains of the residues at positions 2.64 and 7.36 (residue identities for each subtype are shown to on the right). Across all conditions, the M1 mAChR is observed to form a stable hydrogen bond between Y2.64 and E7.36, which stabilizes the open cryptic pocket while the M2 and M4 mAChRs show no such hydrogen bond formation. The M3 mAChR does form a N7.36–Y2.64 hydrogen bond in simulation, but a separate M3-unique interaction between K7.32 and E45.49 keeps the pocket closed to the allosteric site (tracked in Fig. 3 and further highlighted in Supplementary Fig. 9).

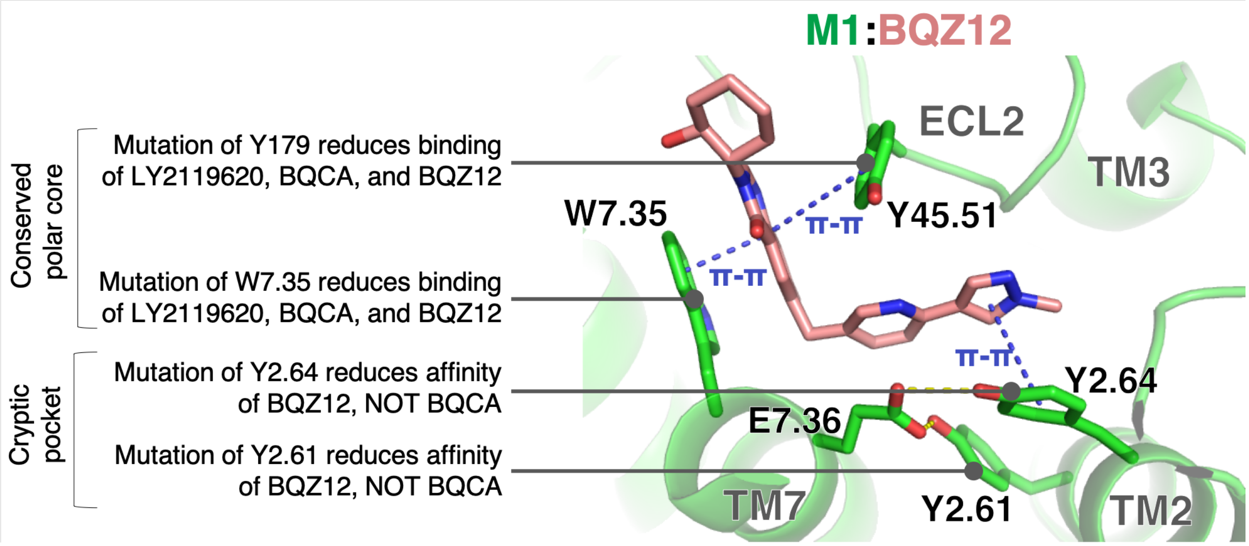

Supplementary Figure 4. The computationally derived binding pose of BQZ12 agrees with all previously published experimental mutagenesis data. Unlike previously proposed poses, our proposed binding pose for BQZ12 (pink), which places the ligand's non-planar arm in the cryptic pocket, agrees with all available experimental data.

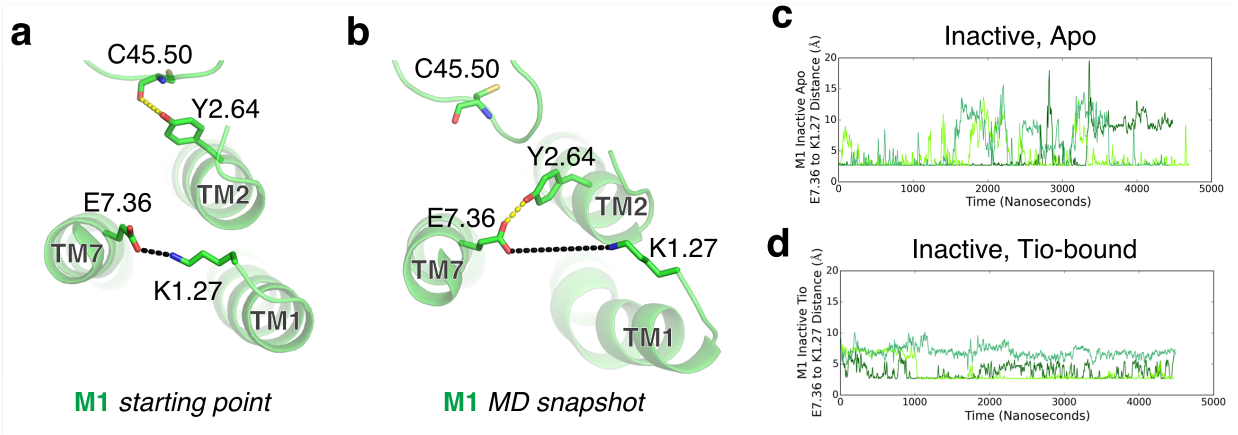

Supplementary Figure 5. E7.36–K1.27 salt bridge breaks spontaneously in inactive-state M1 mAChR simulations. a) In the inactive-state crystal structure of the M1 mAChR, E7.36 forms a salt bridge with K1.27 of TM1 (black line) while Y2.64 forms a hydrogen bond with C45.50 (yellow). **b**) In simulations of the inactive state of the M1 mAChR, the salt bridge between E7.36 and K1.27 is easily and spontaneously broken. Traces of the distance between the K1.27 charged nitrogen and either E7.36 side-chain oxygen are displayed for all Inactive, Apo (**c**) and Inactive, Tio-bound (**d**) simulations. K1.27 was not included in the active state model of the M1 mAChR due to the lack of a corresponding region in the M2 active state mAChR structure used for homology modeling. However, a shift in the position of TM1 in the active state models, which would position K1.27 further away from TM7 than in the inactive state, would make any direct interaction with E7.36 unlikely.

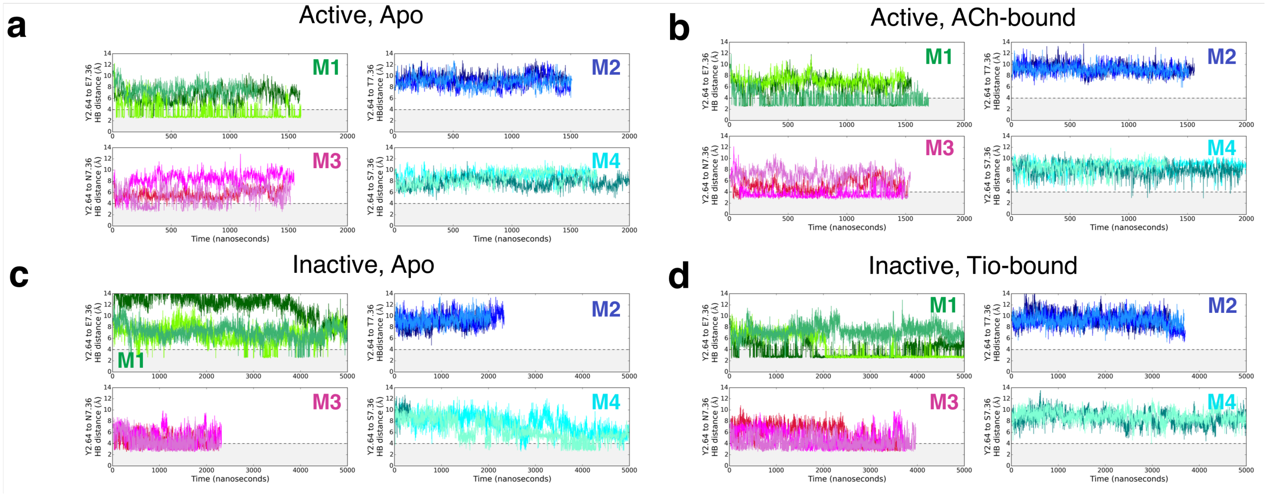

Supplementary Figure 6. The allosteric cryptic pocket opens much more frequently in the M1 mAChR than in the M2, M3, and M4 mAChRs. Traces for the respective distances that track the opening of the cryptic pocket in the mAChR allosteric site, as described in Supplementary Fig. 3, are shown for the following conditions; **a**) Active Apo; **b**) Active ACh-bound; **c**) Inactive Apo; and **d**) Inactive Tio-bound.

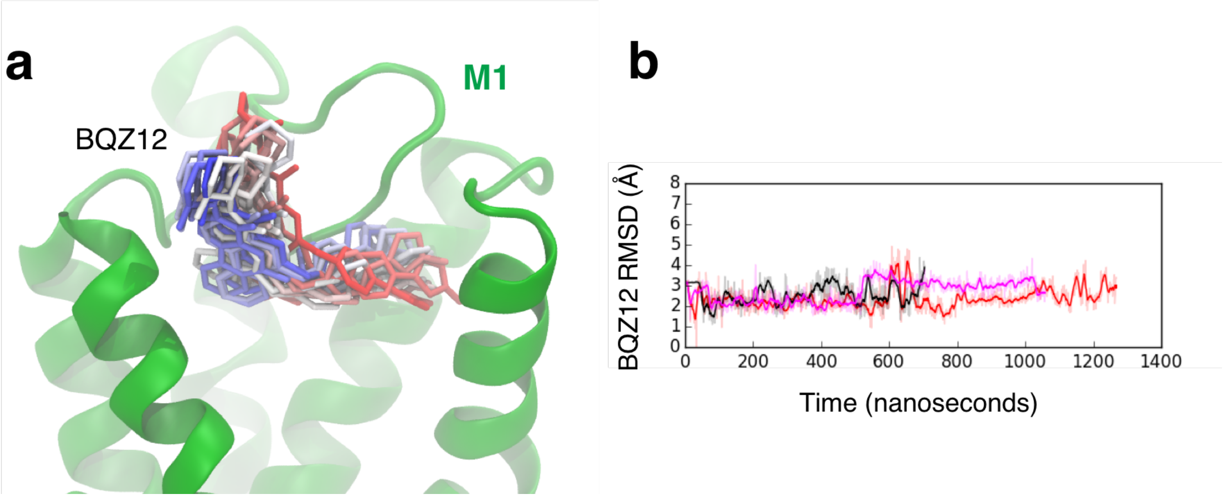

Supplementary Figure 7. BQZ12 remains bound in the cryptic pocket at the allosteric site of the M1 mAChR in simulation. a) Snapshots of BQZ12 taken every 50 nanoseconds (ns) during a 1-microsecond simulation of ACh- and BQZ12-bound active-state M1 mAChR. Ligand snapshots are color coded from red (initial frame) to white to blue (end of simulation). In simulation the nonplanar arm adjusts its position slightly to make a better packing interaction with Y2.64. The aromatic core shifts towards TM6 while still interacting with W7.35 and Y159 (forming the polar core stack observed in the M2–LY2119620 crystal structure). **b)** The RMSD of BQZ12 (heavy atoms only) against an equilibrated pose of BQZ12 in three simulations of ACh- and BQZ12-bound active-state M1 mAChR.

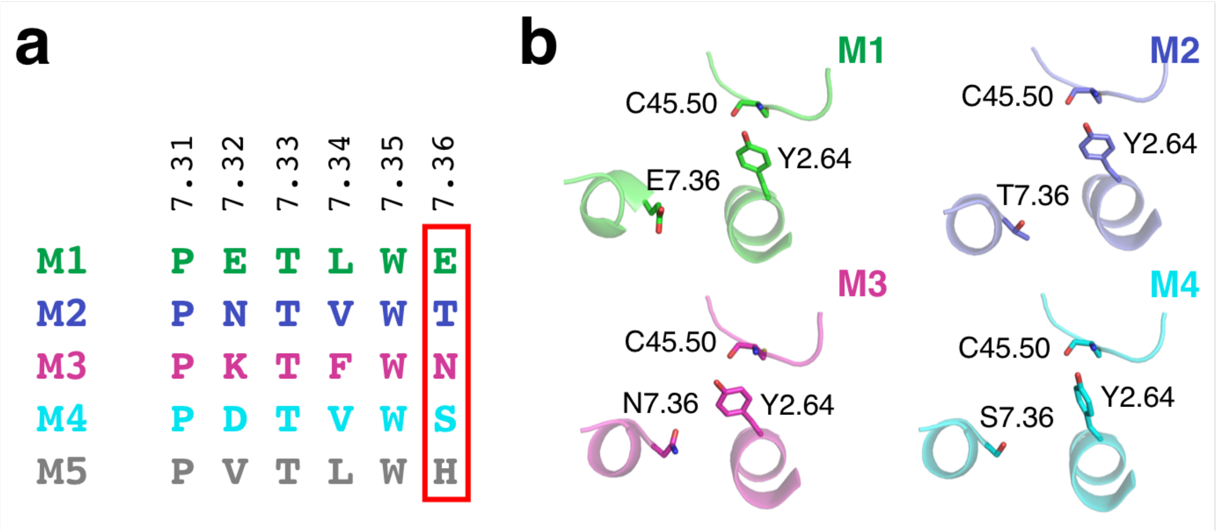

Supplementary Figure 8. Differences in the identity of residue 7.36 may partially explain differences in cryptic pocket stability between mAChR subtypes. a) The residue at position 7.36 is unique at each mAChR subtype. **b**) Inactive state crystal structures of the M1–M4 mAChRs, highlighting residues that play a key role in formation of the cryptic pocket in the M1 mAChR and corresponding residues at other mAChRs. The glutamate at position 7.36 in the M1 mAChR is best suited to stabilize Y2.64 in the orientation required for opening of the cryptic pocket.

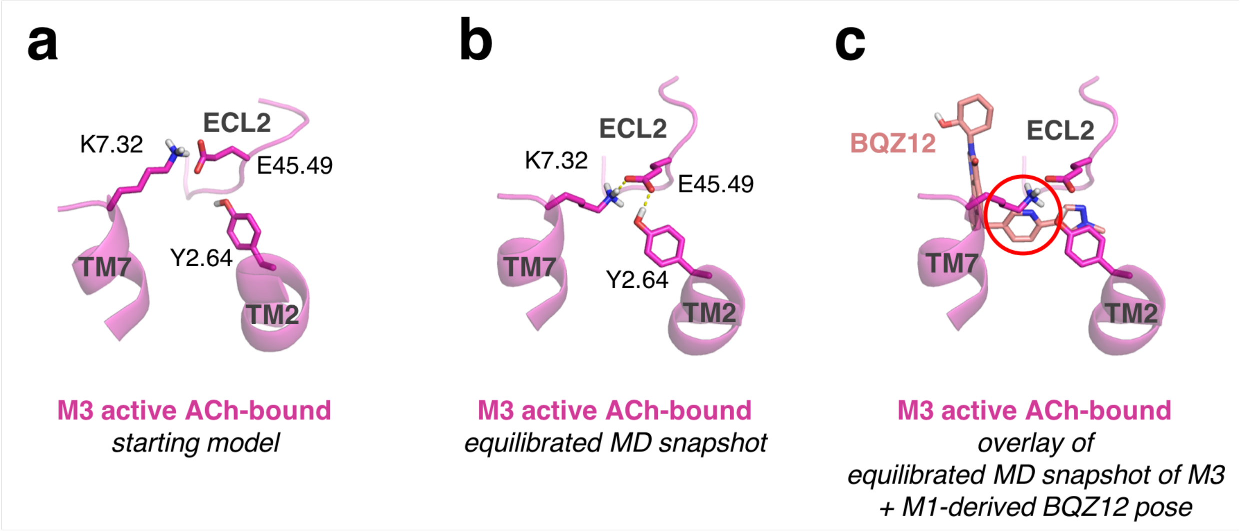

Supplementary Figure 9. The formation of a salt bridge between TM7 and ECL2 in the M3 mAChR also prevents the formation of the cryptic pocket. a) The starting model of the active state of the M3 mAChR based on the active-state structure of the M2 mAChR. **b**) In simulation, K7.32 reorients to form a salt bridge with E220 (E45.49). In this alternative conformation, E220 also forms a second hydrogen bond with Y2.64. This newly formed salt bridge keeps the cryptic pocket closed. **c**) When the M1-derived binding pose of BQZ12 is overlaid with the equilibrated M3 mAChR active structure, the significant clashes between the MD-observed salt bridge and BQZ12 highlight how this BQZ12 binding pose is incompatible with this M3 mAChR conformation. While the snapshots were taken from an active, ACh-bound M3 mAChR simulation, the same interactions were observed in all M3 mAChR simulation conditions.

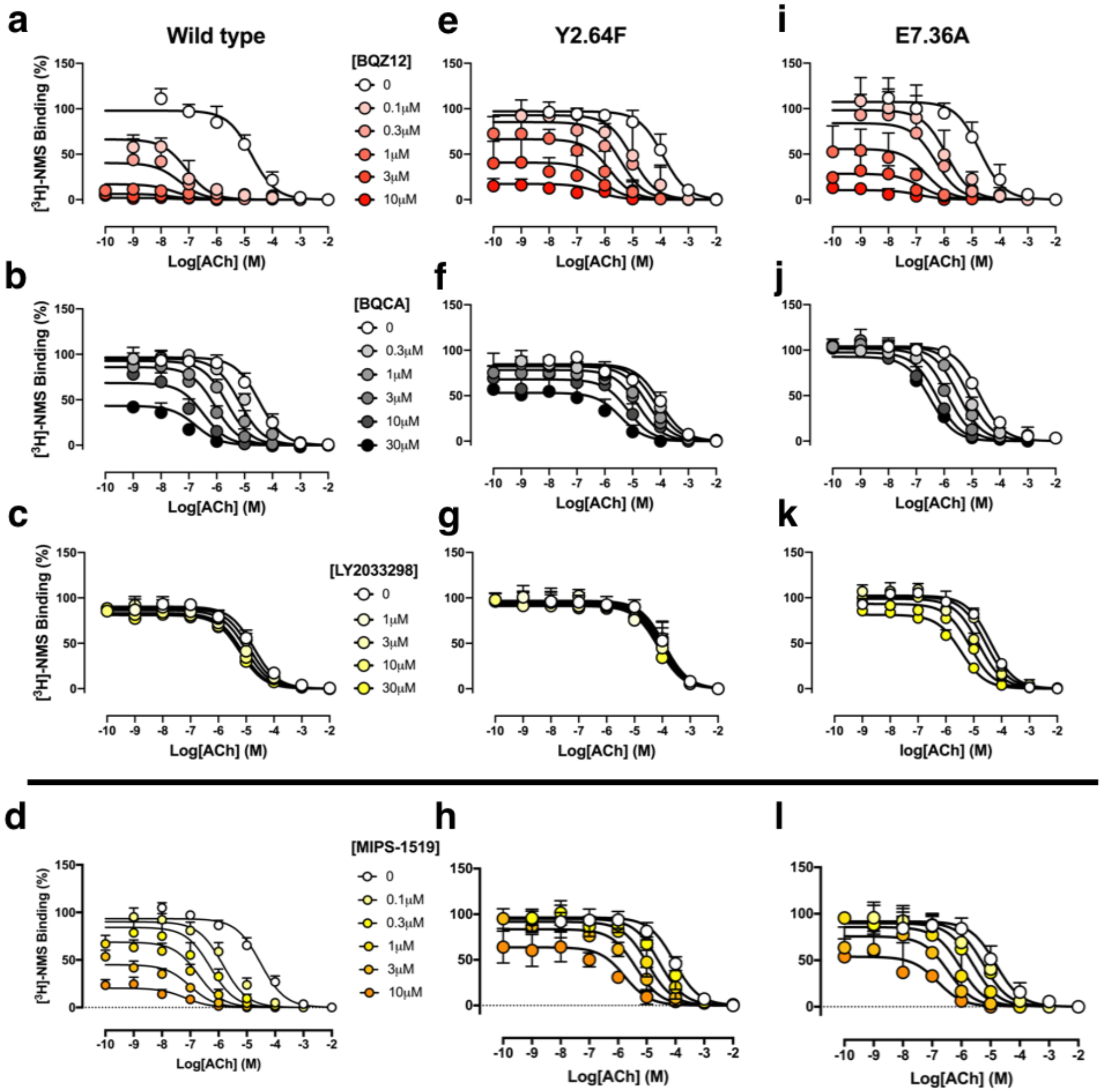

Supplementary Figure 10. Effects of Y2.64F and E7.36A mutations on the binding properties of selected PAMs at the M1 mAChR. Plots show results of [^{3}H]NMS radioligand binding interaction experiments performed between ACh and each modulator at the wild type (**a–d**), Y2.64F (**e–h**) and E7.36 (**i–l**). Data points represent the mean, and error bars the standard error of the mean, across 3 (a, b, c, d, f, i, j, k, and l) or 4 (e, g, and h) independent experiments. Source data are provided in a Source Data file.

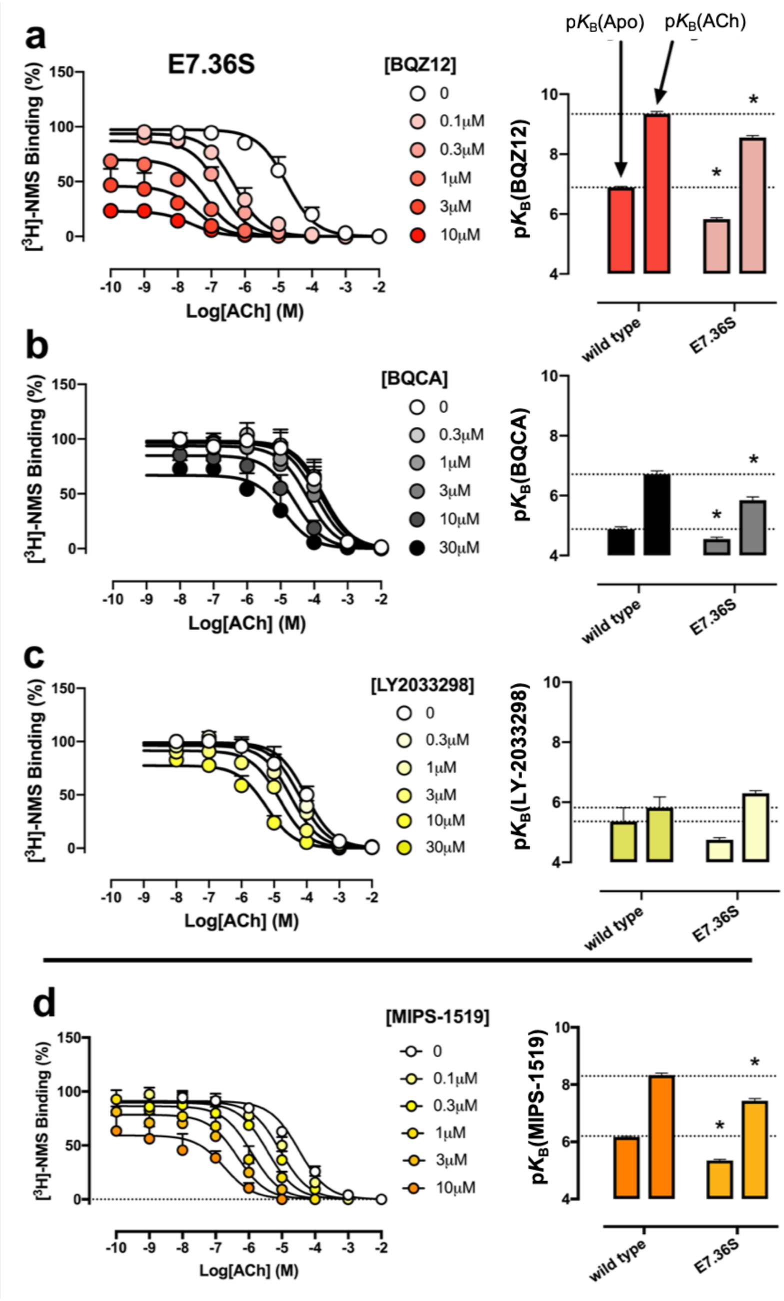

Supplementary Figure 11. Effect of E7.36S mutation on properties of selected modulators at the M1 mAChR. [^{3}H]NMS radioligand binding interaction experiments between ACh and each modulator (**left**), and the respective estimated affinities, pK_B(Apo) and pK_B(ACh) (**right**) for four allosteric modulators: BQZ12 (**a**), BQCA (**b**), LY2033298 (**c**), and MIPS-1519 (**d**). Data points at left represent the mean, and error bars the standard error of the mean, for 3 (MIPS-1519 E7.36S)

or 4 (BQZ12 E7.36S, BQCA E7.36S, LY20333298 E7.36S) independent experiments. Affinity estimates (at right) and their standard errors (shown by error bars) were derived by a global least-squares fit of an allosteric ternary complex model to 3 (BQCA wild type, MIPS-1519 E7.36S), 4 (BQZ12 wild type, BQZ12 E7.36S, BQCA E7.36S, LY20333298 E7.36S, MIPS-1519 wild type), or 6 (LY20333298 wild type) independent experiments, with the constraint that each parameter be shared between the experiments to yield a single best estimate of the parameter and its associated standard error, as derived from the nonlinear regression algorithm. This global pooled analysis approach ensured model convergence in all instances. *, pK_B(Apo) or pK_B(ACh) significantly different (p<0.05) from wild type as determined by one-way ANOVA with Dunnett's post hoc test. Source data are provided in a Source Data file.

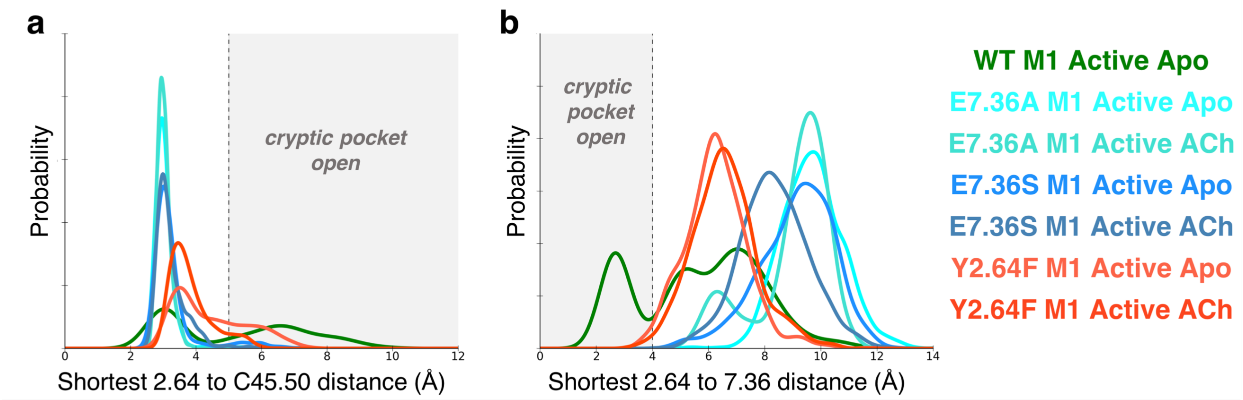

Supplementary Figure 12. Designed mutations prevent opening of the cryptic pocket in simulation. Each of the experimentally generated M1 mAChR mutants in Fig. 4 and Supplementary Fig. 3 were simulated in the active state conformation (both in the presence and absence of ACh). **a)** The distribution of the Y2.64 to C45.50 distance (the measurement highlighted in Fig. 3) is shown for all mutant simulations, with the M1 mAChR Active-Apo reference condition in green. Both the E7.36A and E7.36S mutations resulted in a shift of the distribution towards the closed pocket conformation in the presence or absence of ACh. A similar shift is observed for the Y2.64F mutant, though a small portion of the population still achieves a state where Y2.64 has broken the initial interaction with C45.50. **b)** The distance distributions between 2.64 and 7.36 (as highlighted in Supplementary Fig. 3) are displayed for all mutant simulations, with the M1 mAChR Active-Apo reference condition in green. In all simulations, the disruption of the 2.64–7.36 hydrogen bond in all three mutants disfavor the fully open cryptic pocket conformation.

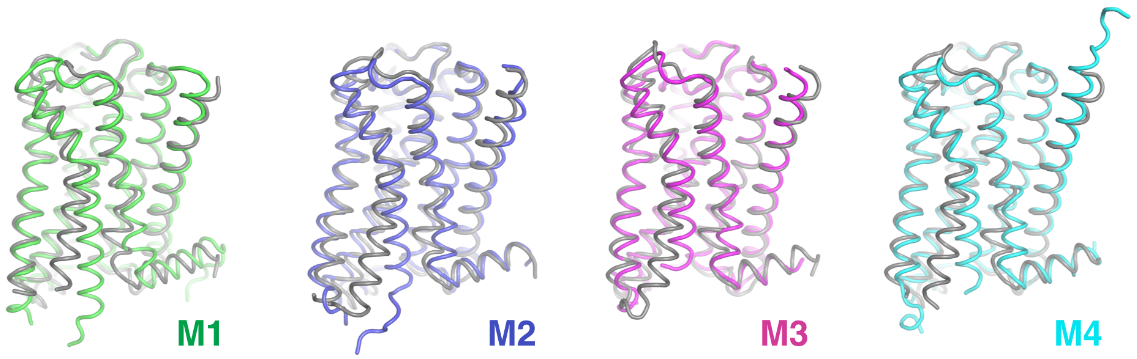

Supplementary Figure 13. Structural alignments of the inactive- and active-state structures of the M1–4 mAChRs used for simulations. Displayed are the inactive-state structures used for simulation for M1–4 (colored as in Figure 1; M1 – green, M2 – blue, M3 – pink, M4 – cyan) with the corresponding subtype active-state structures overlaid in gray. All structures were aligned on the positions of TM1–4 using PyMol.

Supplementary Table 1. PAM affinity estimates for ligand-free and orthosteric ligand-occupied wild-type or mutated mAChRs

	Receptor	pK_B(Apo)[a]	pK_B(NMS)[b]	pK_B(ACh)[c]
BQZ12	M1 wild type	7.09 ± 0.05	=0[d]	9.50 ± 0.13
	M1 Y2.64F	5.86 ± 0.05 *	=0[d]	8.09 ± 0.14 *
	M1 E7.36A	6.38 ± 0.06 *	=0[d]	8.20 ± 0.16 *
	M4 wild type	4.65 ± 0.05	=0[d]	4.71 ± 0.16
BQCA	M1 wild type	5.23 ± 0.05	=0[d]	7.15 ± 0.11
	M1 Y2.64F	4.76 ± 0.23 *	4.15 ± 0.49	6.29 ± 0.17 *
	M1 E7.36A	4.86 ± 0.18 *	4.67 ± 0.20	6.70 ± 0.11 *
	M4 wild type	4.69 ± 0.32	4.49 ± 0.42	5.27 ± 0.22
LY2033298	M1 wild type	5.35 ± 0.22	5.23 ± 0.23	5.83 ± 0.20
	M1 Y2.64F	5.42 ± 0.48	5.38 ± 0.49	5.65 ± 0.46
	M1 E7.36A	4.98 ± 0.22	4.61 ± 0.36	6.28 ± 0.11
	M4 wild type	5.87 ± 0.05	=0[d]	8.12 ± 0.08
MIPS-1519	M1 wild type	6.21 ± 0.06	=0[d]	8.29 ± 0.16
	M1 Y2.64F	4.90 ± 0.05 *	=0[d]	6.89 ± 0.11 *
	M1 E7.36A	5.47 ± 0.107 *	=0[d]	7.22 ± 0.17 *
	M4 wild type	5.38 ± 0.53	5.17 ± 0.64	5.46 ± 0.51

Affinity values for the unoccupied receptor (pK_B(Apo)), the NMS-occupied receptor (pK_B(NMS)), and the ACh-occupied receptor (pK_B(ACh)) are listed for each of the mAChR PAMs investigated

in the study. Affinity values were obtained from analysis of radioligand binding assays performed on intact CHO cells, stably expressing the indicated receptor construct. The M4 mAChR wild-type is included as a control for this set of experiments. Affinity estimates and their standard errors were derived by a global fit of an allosteric ternary complex model to 3 independent experiments for all conditions except BQZ12 Y2.64F, BQZ12 E7.36, LY2033298 Y2.64F, and MIPS-1519 Y2.64F which represent 4 independent experiments, with the constraint that each parameter be shared between the experiments to yield a single best estimate of the parameter and its associated standard error, as derived from the nonlinear regression algorithm. This global pooled analysis approach ensured model convergence in all instances.

a, negative logarithm of the equilibrium dissociation constant of the PAM for the unoccupied receptor.

b, negative logarithm of the equilibrium dissociation constant of the PAM for the [^{3}H]NMS-occupied receptor.

c, negative logarithm of the equilibrium dissociation constant of the PAM for the ACh-occupied receptor.

d, allosteric ligand effect not significantly different (p < 0.0.5; F-test) from complete inhibition of specific radioligand binding, and therefore parameter constrained to 0.

*, pK_B(Apo) or pK_B(ACh) significantly different ($p<0.05$) from wild type as determined by one-way ANOVA with Dunnett's post hoc test.

Supplementary Table 2. Effect of E7.36S mutation in the cryptic pocket of the M1 mAChR on PAM affinity

	Receptor	pK_B(Apo)[a]	pK_B(NMS)[b]	pK_B(ACh)[c]
BQZ12	M1 wild type	6.89 ± 0.03	=0[d]	9.35 ± 0.07
	M1 E7.36S	5.83 ± 0.05 *	4.37 ± 0.33	8.56 ± 0.06 *
BQCA	M1 wild type	4.88 ± 0.08	=0[d]	6.71 ± 0.12
	M1 E7.36S	4.55 ± 0.06 *	=0[d]	5.85 ± 0.11 *
LY2033298	M1 wild type	5.04 ± 0.60	5.29 ± 0.48	5.7 ± 0.66
	M1 E7.36S	4.75 ± 0.07	=0[d]	6.30 ± 0.14
MIPS-1519	M1 wild type	6.17 ± 0.05	=0[d]	8.33 ± 0.14
	M1 E7.36S	5.35 ± 0.07 *	=0[d]	7.43 ± 0.16 *

Affinity values for the unoccupied receptor (pK_B(Apo)), the NMS-occupied receptor (pK_B(NMS)), and the ACh-occupied receptor (pK_B(ACh)) are listed for each of the mAChR PAMs investigated in the study. Affinity values were obtained from analysis of radioligand binding assays performed on intact CHO cells, stably expressing the indicated receptor construct. The receptor constructs in these experiments differ slightly from those of Supplementary Table 1 in that those of Supplementary Table 1 have an N-terminal cmyc tag whereas those listed here do not. Affinity estimates and their standard errors were derived by a global fit of an allosteric ternary complex model to 3 (BQCA wild type, MIPS-1519 E7.36S), 4 (BQZ12 wild type, BQZ12 E7.36S, BQCA E7.36S, LY20333298 E7.36S, MIPS-1519 wild type), or 6 (LY20333298 wild type) independent experiments, with the constraint that each parameter be shared between the experiments to yield a single best estimate of the parameter and its associated standard error, as derived from the

nonlinear regression algorithm. This global pooled analysis approach ensured model convergence in all instances.

a, negative logarithm of the equilibrium dissociation constant of the PAM for the unoccupied receptor.

b, negative logarithm of the equilibrium dissociation constant of the PAM for the [^{3}H]NMS-occupied receptor.

c, negative logarithm of the equilibrium dissociation constant of the PAM for the ACh-occupied receptor.

d, allosteric ligand effect not significantly different (p < 0.0.5; F-test) from complete inhibition of specific radioligand binding, and therefore parameter constrained to 0.

*, pK_B(Apo) or pK_B(ACh) significantly different (p<0.05) from wild type as determined by one-way ANOVA with Dunnett's post hoc test.

Supplementary Table 3. Simulation conditions and lengths

Simulation Condition*	System Dimensions (Å)	Number of Atoms	Simulation Lengths (μs)**	Use of HMR in Simulation
**M1: Inactive, Apo**	85 x 85 x 74	58,731	4.56, 4.70, 4.51	No
**M1: Inactive, Tio-bound**	85 x 85 x 74	58,641	4.56, 4.48, 4.50	No
**M1: Active, Apo**	79 x 76 x 82	47,238	1.63, 1.61, 1.25	Yes
**M1: Active, ACh-bound**	84 x 77 x 82	50,779	1.56, 1.51, 1.70	Yes
**M1: Active, Apo (D2.50 & D3.49 protonated)**	84 x 77 x 82	50,757	2.04, 2.05, 2.03	Yes
**M1: Active, ACh (D2.50 & D3.49 protonated)**	84 x 77 x 82	50,785	2.05, 2.04, 2.02	Yes
**M1 E7.36A: Active, Apo**	84 x 77 x 82	50,748	1.59, 1.52, 1.51	Yes
**M1 E7.36A: Active, ACh-bound**	84 x 77 x 82	50,776	1.54, 1.54, 1.54	Yes
**M1 E7.36S: Active, Apo**	84 x 77 x 82	50,749	1.58, 1.58, 1.51	Yes
**M1 E7.36S: Active, ACh-bound**	84 x 77 x 82	50,777	1.52, 1.50, 1.53	Yes
**M1 Y2.64F: Active, Apo**	84 x 77 x 82	50,750	1.54, 1.51, 1.52	Yes
**M1 Y2.64F: Active, ACh-bound**	84 x 77 82	50,778	1.52, 1.51, 1.52	Yes
**M1: Active, ACh & BQZ12-bound**	84 x 77 x 82	50,742	1.27, 1.29, 1.06	Yes
**M2: Inactive, Apo**	82 x 78 x 84	51,614	1.08, 1.20, 1.06	No
**M2: Inactive, Tio-bound**	82 x 78 x 74	51,664	1.78, 1.93, 1.92	No
**M2: Active, Apo**	85 x 76 x 82	50,998	1.50, 1.51, 1.52	Yes
**M2: Active, ACh-bound**	85 x 76 x 82	50,538	1.56, 1.51, 1.52	Yes
**M3: Inactive, Apo**	83 x 74 x 80	47,916	1.18, 1.20, 1.18	No
**M3: Inactive, Tio-bound**	83 x 74 x 70	47,963	1.90, 1.95, 2.06	No
**M3: Active, Apo**	87 x 76 x 83	53,565	1.46, 1.55, 1.52	Yes

**M3: Active, ACh-bound**	87 x 76 x 81	52,220	1.52, 1.55, 1.54	Yes
**M4: Inactive, Apo**	83 x 83 x 90	59,176	6.65, 6.71, 6.78	No
**M4: Inactive, Tio-bound**	82 x 82 x 90	59,016	7.08, 6.29, 6.23	No
**M4: Active, Apo**	84 x 84 x 76	57,892	1.78, 2.17, 1.76	No
**M4: Active, ACh-bound**	87 x 87 x 76	63,192	2.17, 2.00, 1.36	No

* - Unless otherwise noted, M1 and M3 simulations had D3.49 and D2.50 unprotonated while M2 and M4 simulations had D3.49 and D2.50 protonated.

** - Each entry represents an independent simulation.
